# A modified oblique lumbar interbody fusion: A better way to establish an exposure under direct microscopic vision

**DOI:** 10.3389/fsurg.2023.1130489

**Published:** 2023-03-06

**Authors:** Kai Wang, Xiangyu Zhang, Zirun Zhao, Dean Chou, Fengzeng Jian, Hao Wu

**Affiliations:** ^1^ Department of Neurosurgery, Xuanwu Hospital of Capital Medical University, Beijing, China; ^2^ Department of Radiology, Renaissance School of Medicine, Stony Brook University, Stony Brook, NY, United States; ^3^ Department of Neurosurgery, University of California San Francisco, San Francisco, CA, United States

**Keywords:** oblique lumbar interbody fusion, psoas, direct microscopic vision, nerve injury, vascular injury

## Abstract

**Study design:**

This is a retrospective study.

**Objective:**

To demonstrate a modified oblique lumbar interbody fusion (OILF) technique for L1–L5.

**Methods:**

The modified technique splits anterior portion of psoas belly to access the oblique corridor (OC) anteroinferior to psoas, minimizing psoas manipulation and retraction and avoiding nerve injury while offering excellent microscopic visualization. Psoas weakness and neurovascular complication rates in patients treated with traditional OLIF (T-OLIF) or anteroinferior psoas OLIF (AP-OLIF) were retrospectively reviewed. Clinical outcomes were also reviewed.

**Results:**

A total of 162 cases treated with T-OLIF (*n* = 73) and AP-OLIF (*n* = 89) for degenerative lumbar disease were included. The mean operative time and blood loss were less with AP-OLIF (*P* < 0.01). Approach related complications were 14 (19.1%) with T-OLIF and 4 (4.5%) with AP-OLIF. Postoperative visual analog scale (VAS) and Oswestry Disability Index (ODI) scores improved in both T-OIF and AP-OIF groups (*P* < 0.01).

**Conclusion:**

The modified OLIF technique (AP-OLIF) is characterized by an easy exposure of the lumbar spine under direct microscopic vision, resulting in less psoas weakness and neurovascular injury.

## Introduction

In recent years, retroperitoneal lateral lumbar interbody fusion has become a popular technique for treating lumbar degenerative disease. Advantages are its clinical efficacy, ease of access, larger cage placement, minimally invasive nature, and faster patient recovery (1–5). The minimally invasive lateral transpsoas approach to the lumbar spine, known as extreme lateral interbody fusion or direct lateral interbody fusion, was first described in 2001 (1, 2). However, the transpsoas approach is associated with direct muscle injury and a risk of injury to the lumbar plexus because the trajectory courses through the psoas (6–8). The oblique lumbar interbody fusion (OLIF) accesses the spine between the great vessels and psoas muscle, allowing for psoas preservation and avoiding the lumbar plexus injury. It was introduced as an alternative procedure to the transpsoas lateral lumbar interbody fusion (3, 4).

**Figure 1:**
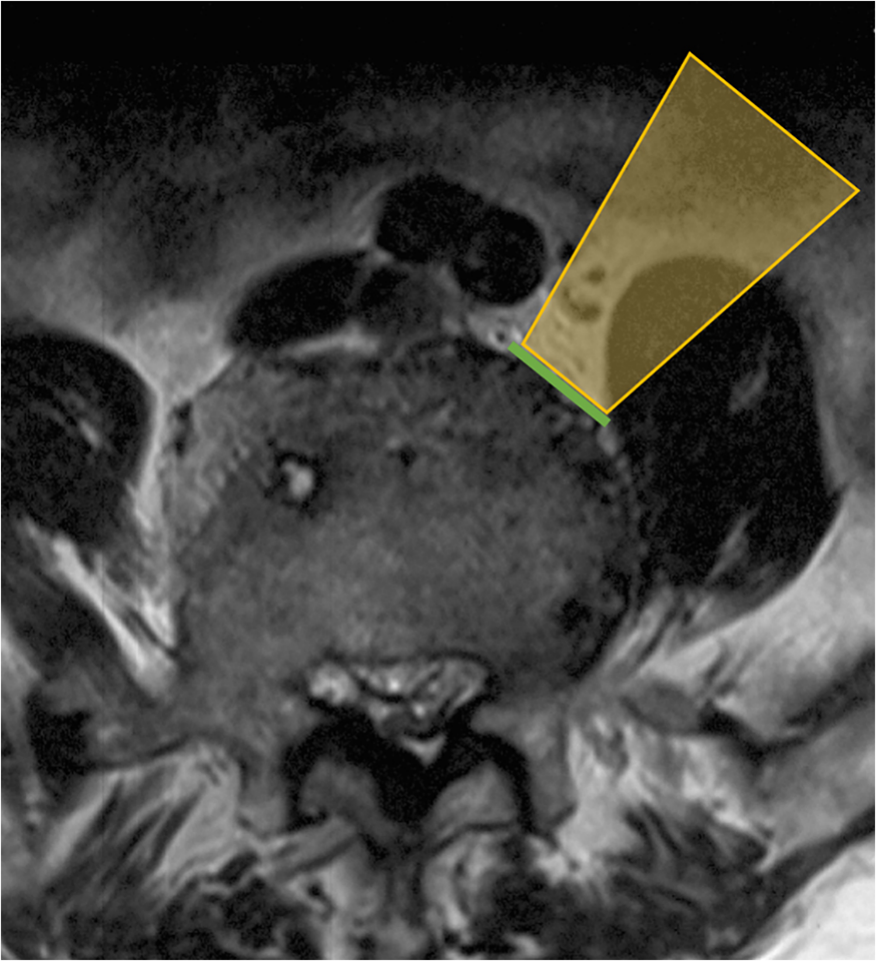
Ideal oblique corridor accessing to intervertebral disc may be blocked by hypertrophic belly of psoas (note the orange area). Green line indicates ideal OLIF corridor).

Safe surgical exposure is the core of the OLIF approach. According to one anatomic study, the mean access corridor diameters at L1–L5 were 15.0–20.58 mm (9, 10). Radiographic studies have reported the access corridor diameter at L2–L5 were 10.28–16.04 mm and decrease further from supine to the right lateral decubitus position due to a relaxed psoas (11–13). However, the width of the cages commonly used were 18 and 22 mm, and the width of the retractor blades were 22 and 25 mm. Both the widths of cages and of the blades can be larger than the diameter of the oblique corridor itself. Nevertheless, with mild psoas retraction, the corridor can reach 19.67–28.00 mm (9, 10). However, psoas retraction and over manipulation can cause lumbar plexus damage and ipsilateral iliopsoas weakness, which is counter to the intention of the OLIF (14–19). Moreover, the lumbar sympathetic trunk (LST) may be identified in this longitudinal corridor and may require manipulation during the operation (20). Segmental vessels also traverse this region in a medial to lateral direction. Thus, introducing probe and dilator without direct visualization may endanger LST and segmental vessels. To avoid these issues, the anterior to the psoas (ATP) approach was developed (3, 15). However, the psoas is a bulky structure, and a large psoas in some patients may block the corridor to intervertebral disc. This anatomic reality sometimes makes it difficult to establish proper exposure and inevitably may require significant retraction of psoas, increasing the risk of neurovascular injury (Figure 1) (21).

**Figure 2:**
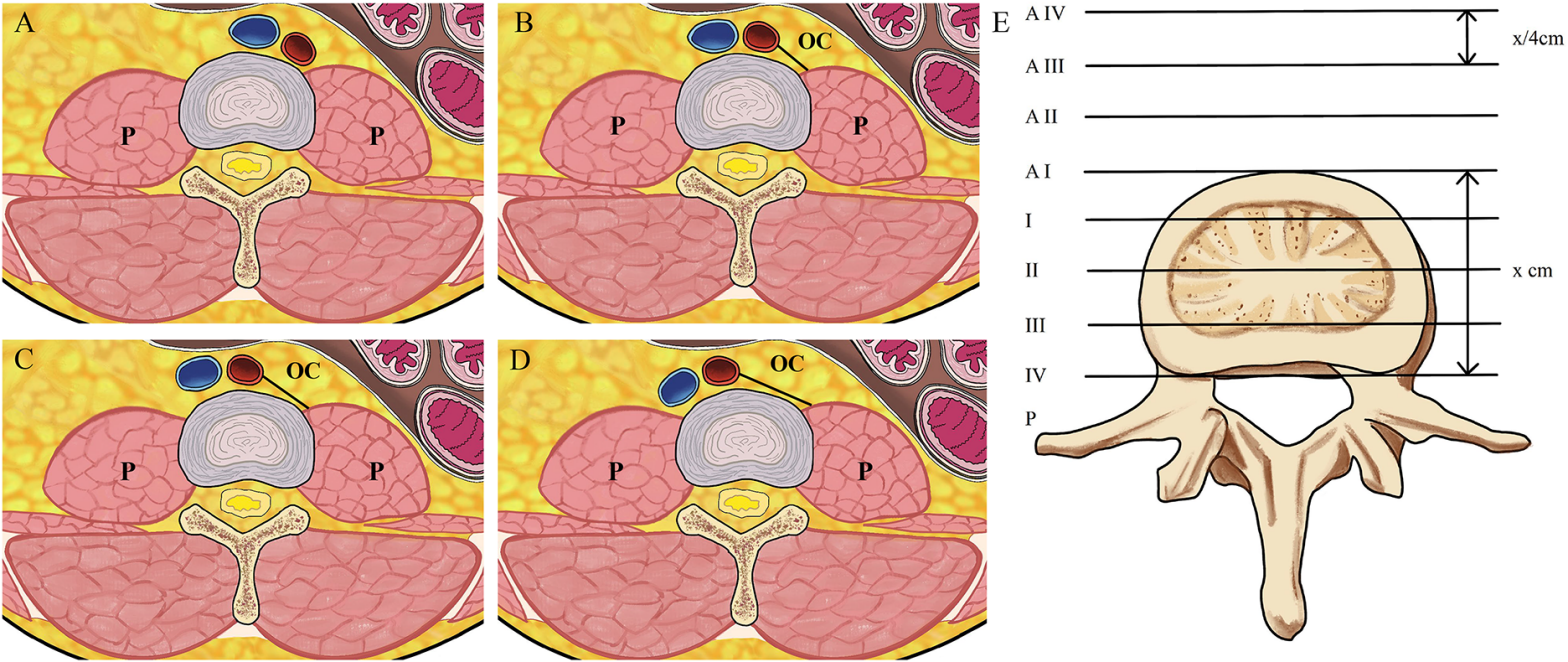
(**A**) OC grade 0, that is, no measurable corridor; (**B**) OC grade 1 (OC ≤ 1 cm); (**C**) OC grade 2, (1 cm < OC ≤ 2 cm); (**D**) OC grade 3 (1 cm < OC ≤ 2 cm); (**E**) the modified Moro’s system is explained. The anterior zone refers to the area anterior to the anterior border of the disc; the posterior zone is the anatomical opposite. Zones I–IV are equally divided zones of the disc in the anteriorposterior diameter. The anterior zone is further subdivided into four zones named AI–AIV, with intervals one-quarter (x/4 cm) the AP length of the disc (x cm), reflecting a mirror image of the original zones I–IV. P indicates psoas, OC indicates oblique corridor.

To define the location and shape of psoas, Moro et al. classified the location of the anterior border of psoas major in one of six zones. Ng et al. modified the Moro system to incorporate classifications of difficult psoas anatomy, that is, high-rising psoas/teardrop psoas/Mickey-mouse psoas (22). The modification further subdivides the anterior zone into four zones named AI–AIV, reflecting a mirror image of the zones I–IV with intervals calculated in quarters respective to the IV disc length as in the original Moro’s classification. Based on the radiographic anatomical study, Ng et al. proposed that having an oblique corridor (OC) grade of 0 or a high riding psoas (Modified Moro’s AII, AIII or AIV) precluded the OLIF approach (Figure 2) (22).

In order to address these issues, we have developed the anteroinferior psoas OLIF (AP-OLIF) approach to L1–L5. This approach establishes an easy exposure under direct microscopic vision.

## Methods

This is a retrospective study. Informed consent was obtained from each patient.

### Surgical technique

#### Approach selection indication

For patients undergoing surgery for degenerative lumbar conditions, the lumbar axial MRIs were preoperatively evaluated. Psoas was graded using previously published criteria (22). T-OLIF was used for patients with grade 3 OC or for psoas muscles with the modified Moro classification of types II, III, or IV. Patients with grade 0, 1 or 2 OC or with modified Moro classification of I and AI–AIV were treated with AP-OLIF.

#### Positioning

**Figure 3:**
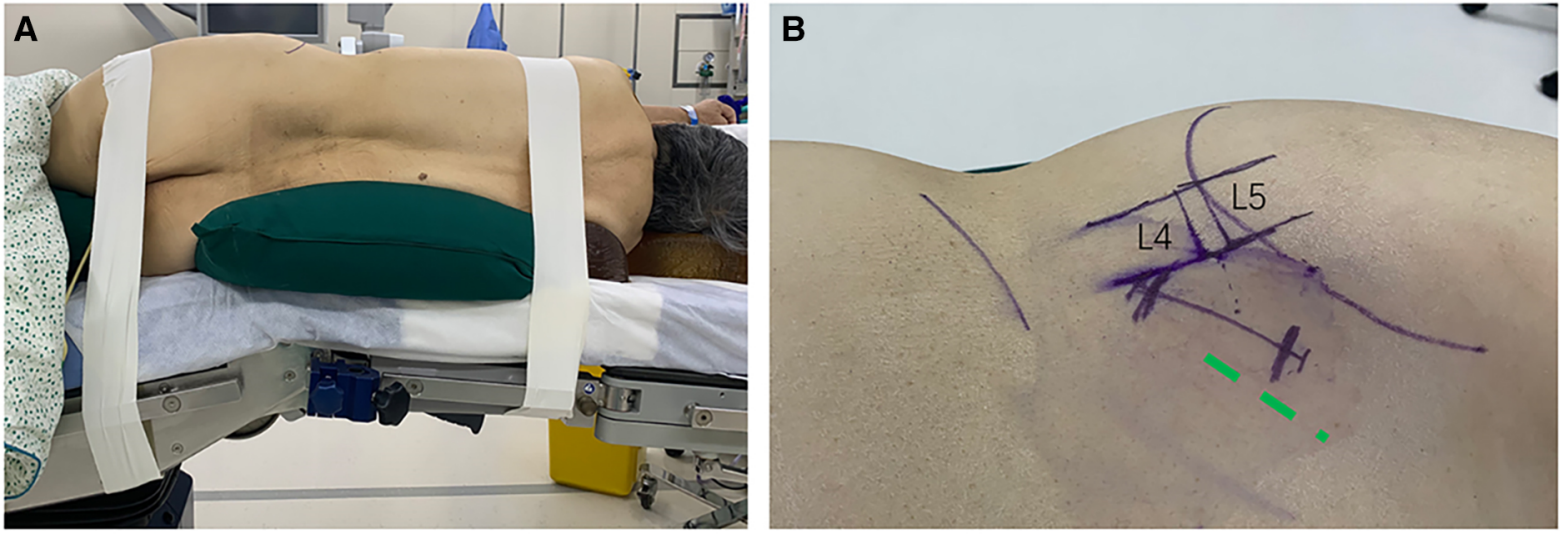
(**A**) Lateral positioning with a break in the table. (**B**) Skin marks; Green dotted line indicates the incision of T-OLIF.

The patient is placed into the left or right lateral decubitus position on a radiolucent table and properly secured to the table with adhesive tape at the axillary and pelvic areas (Figure 3). The convex side of the deformity is preferred for the surgical approach in spinal deformity cases (23). The hip is positioned just below the table break and is gently flexed to relax the psoas muscle and femoral nerve. A pillow is placed under the flank to stabilize the spine and to allow gentle breaking of the table. A slight break can be helpful to stretch the skin and get more space between the iliac crest and the rib cage.

#### Incision planning

Fluoroscopy is utilized to mark the anterior and posterior margins of the vertebral body as well as the target disc space. The skin incision is more lateral compared to the typical OLIF incision (Figure 3). It should start at the anterior margin of the vertebral body and slope obliquely in the line of the external oblique muscle fibers. If a single level is involved, the center of the incision is at the disc level. If two levels are addressed, the center of the incision is between the two target discs. A single or double level surgery can comfortably be done using a 50–60 mm skin incision. Three levels require a longer skin incision, and four levels are better done with two parallel small incisions.

#### Exposure

**Figure 4:**
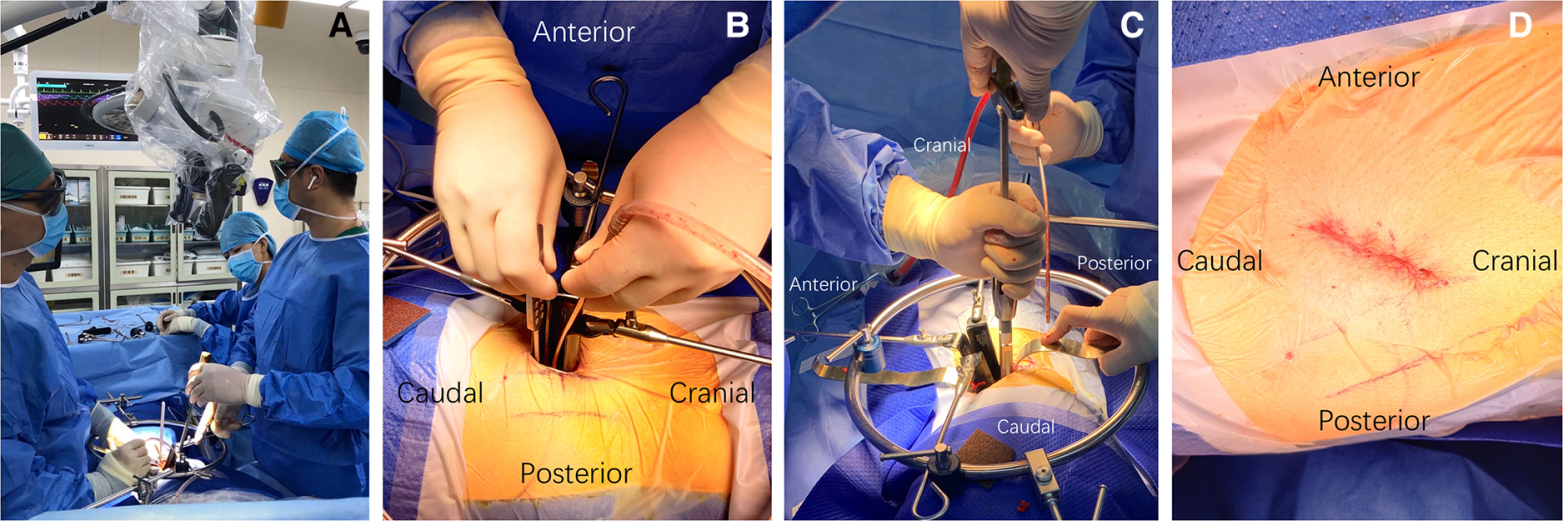
(**A**) Dissecting and achieving stable exposure under direct microscopic vision; (**B**) incising annulus fibrosus; (**C**) AP-OLIF technique allows for inserting a cage perpendicular to the spine; (**D**) surgical wound after closure.

The flank is prepared and draped in the usual sterile fashion. The iliac crest should be reserved for bone marrow aspirating. Following the skin incision, the external oblique fascia is cut, and the 3 abdominal muscles are bluntly dissected in the lines of their fibers. The transversalis fascia is opened from posterior to anterior to avoid injury to the peritoneum. Then the retroperitoneal fat is identified, and the peritoneum is swept anteriorly using a Deaver retractor to expose the belly of the psoas under direct visualization of microscope (Mitaka, Kestrel View) (Figure 4). The psoas is a major intra-abdominal landmark which can be reliably identified throughout the procedure.

**Figure 5:**
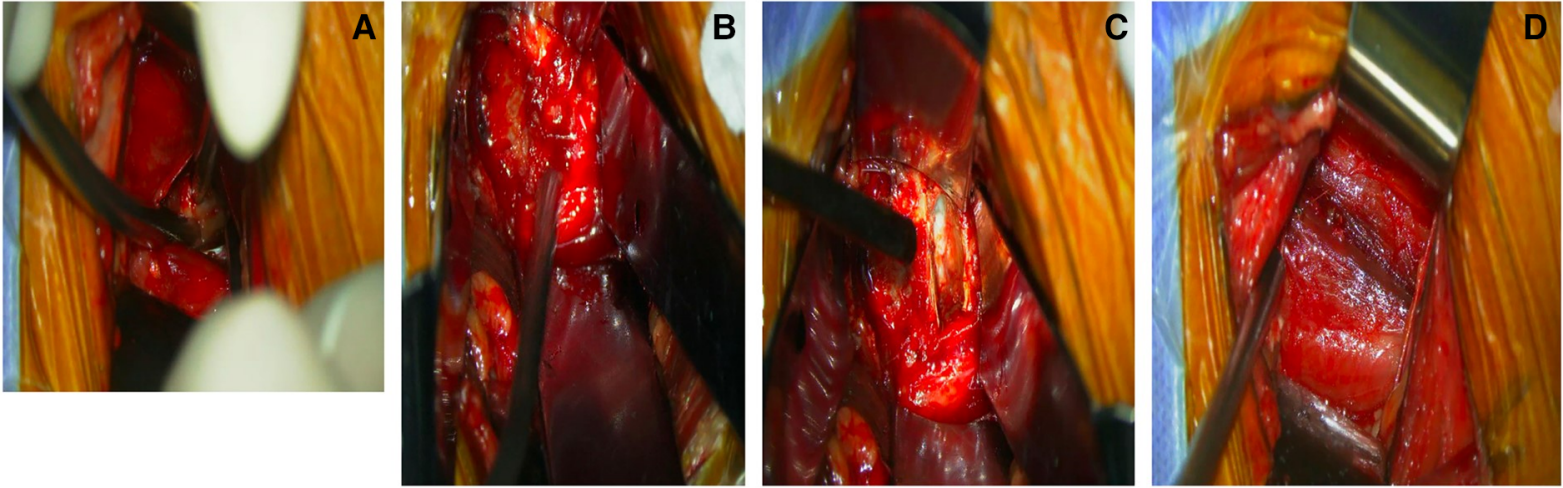
(**A**) Intraoperative photograph showing manual psoas dissection under direct microscopic vision; (**B**) intraoperative photograph showing wide and safe access can be achieved with AP-OLIF approach; (**C**) discectomy under microscopic vision. (**D**) Intact psoas after manipulation.

**Figure 6:**
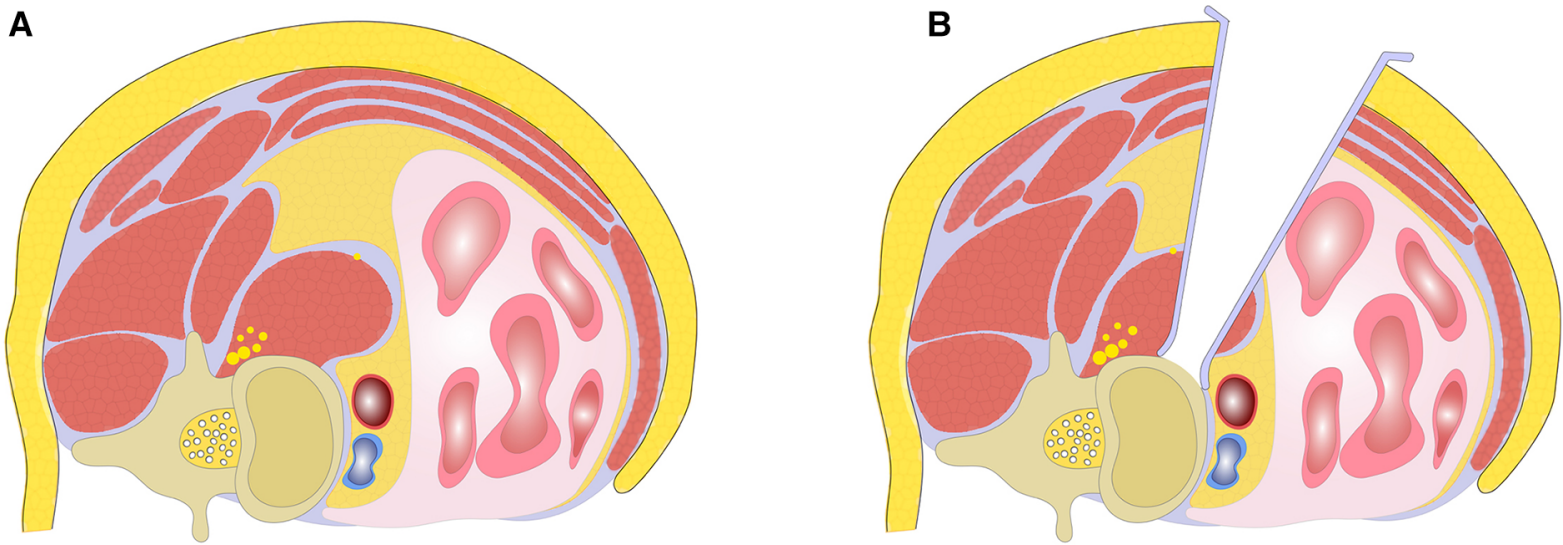
(**A**) Illustration of teardrop psoas blocking the oblique corridor; (**B**) achieving stable exposure with AP-OLIF technique by splitting anterior portion of psoas.

The anterior portion of psoas belly is bluntly separated at the level of the disc space in the direction of its fibers under direct microscopic visualization. Usually up to a quarter of the psoas major muscle needs to be dissected. The dissection is carried out in an anteroinferior manner to expose the underlying disc (Figure 5). After fluoroscopic confirmation of the target level, adjustable and blunt-tip retractor blades are placed to maintain the exposure (SynFrame retractor system, Depuy). A 90° lateral access to the disc space is gained (Figure 6). The dissection plane should be ventral to the neural elements throughout the course of the operation (Figure 6). The genitofemoral nerve on anterior surface of psoas should be carefully identified and gently retracted dorsally with psoas. If a traversing nerve of lumbar plexus is encountered during the procedure, it should be gently retracted dorsally. If more than one disc needs to be addressed, a similar maneuver of psoas splitting and exposure can be carried out. Uninterrupted splitting psoas longitudinally through multiple disc levels should be avoided, in order to excessive manipulation of the psoas and lumbar plexus.

#### Discectomy, endplate preparation and cage insertion

**Figure 7:**
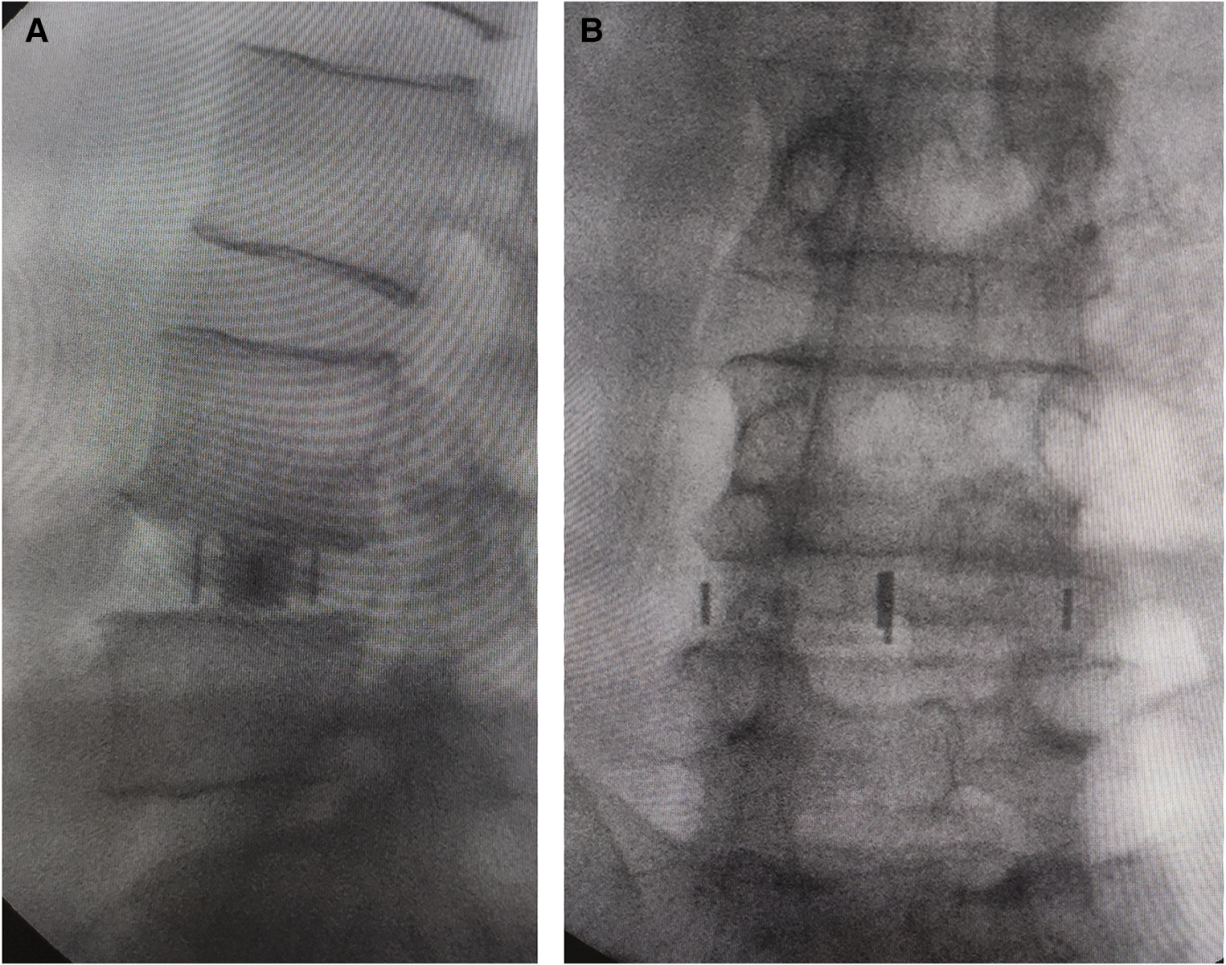
(**A**) Intraoperative anteroposterior and (**B**) lateral radiographs to confirm the ideal position of the cage. Anteroposterior and lateral metal markers show the cage was almost perpendicular to the spine. (**A**) Anteroposterior metal markers show the cage extending the entire width of the disc and vertebral body with support from the lateral aspect of the vertebral body bilaterally.

The discectomy is performed with attention not to violate the vertebral endplates. Direct decompression of the thecal sac and contralateral foramen by resecting posterior disc and osteophyte can be achieved under microscopic vision (Figures 4, 5). If the disc space is more severely collapsed, stepwise distraction with different sized trials and frequent radiographic control imaging is needed to avoid violation of the end plates. The appropriate sized interbody cage can be determined based on intraoperative trial sizing. The position of the trials should be confirmed by fluoroscopy. A 50 or 55 mm length cage is usually selected to span the vertebral body and rest on the dense apophyseal ring bilaterally to minimize cage subsidence (Figure 7). Autologous bone marrow is aspirated from the iliac crest and mixed with allograft and bone morphogenetic protein (BMP). An appropriately sized cage is packed with graft mixture and placed into the disc space in a relatively vertical direction. The ideal position of the cage is confirmed by fluoroscopy (Figure 7). After placement of the cage, inspection of the retroperitoneal space for any active bleeding or peritoneal violation is performed under direct microscopic visualization. This step is followed by closure of the abdominal muscles and fascia in a layered fashion. The interbody fusion can be supplemented by posterior instrumentation either during the same surgical setting or in a staged fashion.

### Cohort description and data collection

**Figure 8:**
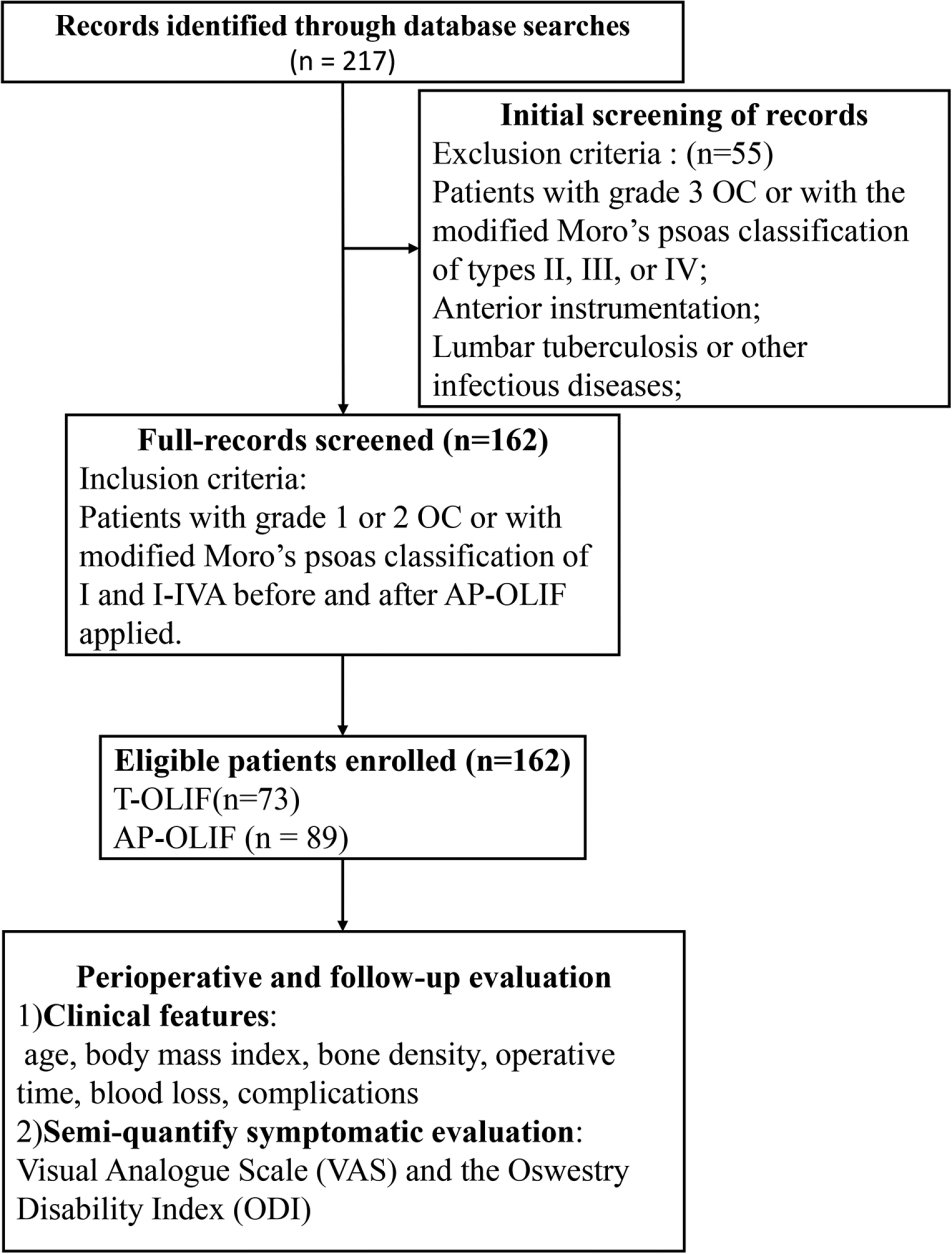
Flow chart of the study selection and tracking process.

Patients who were treated with OLIF for degenerative lumbar conditions in our hospital between October 2016 and September 2019 were retrospectively reviewed. Inclusion criteria were: patients with grade 1 or 2 OC or with modified Moro’s psoas classification of I and AI–AIV before and after AP-OLIF applied. Exclusion criteria were: (1) Patients with grade 3 OC or with the modified Moro’s psoas classification of types II, III, or IV; (2) Patients with anterior instrumentation; (3) Patients with complicated lumbar tumor, lumbar tuberculosis or other infectious diseases, and a history of previous lumbar surgery. Patients eligible for inclusion were divided into two groups based on T-OLIF or AP-OLIF treatment (Figure 8). All the surgeries were performed by one fellowship-trained spine surgeon. No other surgeon (general or vascular) was involved during the exposure of any case. The electronic medical records including inpatient medical records, operative notes, surgical discharge summaries, and clinic visit notes were reviewed by an independent research fellow who was not directly involved in patient care. The collected data included patients’ demographics, primary diagnosis, surgical data (type of operative procedure, levels of fusion, operative time and blood loss), and complications related to the two different surgical approaches (psoas weakness, neurological injury, vascular injury and ureteral injury). These data points were selected in accordance with the findings of previous reports (15, 17–19). Back and leg pain was evaluated according to the visual analog scale (VAS). The Oswestry Disability Index (ODI) at preoperative and 1-year postoperative follow-up was also compared.

### Statistical analysis

Statistics were calculated using SPSS 23 statistics software. All measurements were described with percentages or mean values with standard error of the means. The patients’ demographic and clinical results were compared with the student’s *t*-test, and the approach-related complications were compared with the chi-square test.

## Results

A total of 217 operative patients were reviewed and of those, 162 (78.3%) cases met the inclusion criteria. All the enrolled patients had grade 1 or 2 OC or with modified Moro’s psoas classification of I and I–IVA and were treated by OLIF. Of the 162 patients included, 73 (45.1%) patients were treated with T-OLIF and 89 (54.9%) were treated with AP-OLIF treatment. Patients’ demographics are shown in Table 1. There were no significant differences in age, BMI and levels of fusion between the two groups (*P* > 0.05). The mean operative times (102.5 ± 56.8 vs. 186.3 ± 120.0 min, *P* < 0.01) and blood loss (49.7 ± 102.1 vs. 143.5 ± 225.2 ml, *P* < 0.01) with AP-OLIF were significantly less than T-OLIF. Approach related complications were 14 (19.1%) with T-OLIF and 4 (4.5%) with AP-OLIF. A chi-square test showed a reduction in procedural risks with AP-OLIF (*P* = 0.03). Detailed approach related complications are shown in Table 2. Postoperative VAS and ODI scores were improved in both T-OLIF and AP-OLIF groups (*P* < 0.01) (Table 3).

**Table 1:** Patient demographics.

	Current series (T-OLIF)	Current series (AP-OLIF)	*P* value*
Total number of patients (male, female)	73 (M: 35, F: 38)	89 (M: 33, F: 56)	
Age (in years)	67.5 ± 9.8	66.6 ± 9.5	0.62
BMI (kg/m^2^)	25.7 ± 3.7	25.8 ± 3.9	0.39
Levels of fusion	1.8 ± 0.9	2.0 ± 0.9	0.24
Operation time (min)	186.3 ± 120.0	102.5 ± 56.8	<0.01
Blood loss (ml)	143.5 ± 225.2	49.7 ± 102.1	<0.01
Average follow-up time (month)	35.4 ± 4.7	23.9 ± 2.2	

Diagnosis (some patient had multiple diagnosis).

* *P* value <0.05 considered as significant.

**Table 2:** Comparison of approach related complications rate of current series with other reports.

		Current series (T-OLIF)	Current series (AP-OLIF)	Fujibayashi et al.	Abe et al.	Zeng et al.	Tannoury et al.
Neurological injury	Sensory nerve injury	5.5%	1.1%	3.5%	13.5% (including motor nerve injury and psoas weakness, Transient)	2.98%	3.1% (Persistent)
	Motor nerve injury	2.7%	-	1%	-	1.28%	0.95%
	Sympathetic trunk injury	1.4%	-	-	-	1.28%	
	Spinal nerve injury	-	-	-	0.6%	0.43%	
	Cauda equina injury	-	-	-	0.6%	-	
	Retrograde ejaculation	-	-	-	-	-	0.2%
Psoas weakness		5.5%	2.2%	3.0%	-	3.83%	0.5%
Vascular injury	Major vascular injury	1.4%	-	0.1%	-	0.43%	0.1%
	Segmental vascular injury	2.7%	1.1%	0.7%	2.6%	1.7%	0.2%
	Other vessels	-	-	-	1.3%	0.86%	0.1%
	Hematoma	1.4%	-	0.3%	-	-	0.1%
Ureteral injury		-	-	0.3%	0.6%	-	-

**Table 3:** Clinical outcomes.

Parameter	Value preop	Value postop	*P* value*
**T-OLIF**			
VAS, back	5.1 ± 1.8	2.7 ± 1.3	<0.01
VAS, leg	3.9 ± 2.1	1.7 ± 1.2	<0.01
ODI	30.7 ± 14.7	15.5 ± 8.4	<0.01
**AP-OLIF**			
VAS, back	4.5 ± 1.6	1.9 ± 0.7	<0.01
VAS, leg	3.5 ± 1.5	1.4 ± 0.7	<0.01
ODI	40.6 ± 12.8	13.3 ± 5.0	<0.01

* *P* value < 0.05 considered as significant.

## Discussion

The lateral transpsoas approach was introduced with several unique advantages (1, 2). This technique can provide increased stability, allow for effective indirect decompression of the neural elements, and have dramatic effects on coronal and sagittal alignment. However, the main limitation of the lateral transpsoas approach is the potential injury of the lumbar plexus given that the psoas is directly transgressed (6, 7, 15, 24). Another disadvantage to the transpsoas approach is the inability to access the L5–S1 disc space due to the position of the iliac crest (9, 10). The oblique corridor between the anterior great vessels and the psoas muscles was introduced and considered to decreased risk of neurologic deficits and allow for easier access to L4/5 (3, 4). However, complications such as nerve injury, iliopsoas weakness and vascular injury were still reported (4, 15, 17–19). In order to reduce psoas weakness and neurovascular complications, we modified the T-OLIF technique, allowing for easy exposure through direct microscopic visualization during the procedure. Our results showed that the modified AP-OLIF technique can achieve a satisfactory clinical outcomes while reducing approach-related psoas weakness and neurovascular complications (Tables 2, 3).

Although an oblique approach to the disc space appears logical, an oblique insertional direction uses shorter cages to avoid contralateral neural injury (16). Silvestre et al. who coined the term “OLIF” used a banana shaped cage and recommended cages shorter than 30 mm to avoid injury to the contralateral traversing nerve root (4). Subsequently other options were introduced that combined the oblique approach with use of larger lateral cages. This technique introduced in 2012 allowed for an oblique approach and subsequently orthogonally placing the lateral cages in a true lateral position. Angled inserters and instruments were developed to place large lateral cages at 90° to the disc space *via* an oblique approach (15, 16). We use a more lateral OLIF incision (Figure 3), allowing us to establish a trajectory as orthogonal as possible and place large lateral interbody cages at approximately 90° to the disc space with a special straight inserter (Figure 4). Moreover, an orthogonal trajectory allows us to place the cage at a preferable position, as an anteriorly placed cage gives more lordosis while a posteriorly placed cage results in more distraction and indirect decompression.

The OLIF has a small, deep incision that makes it difficult to expose and visualize spine column. The original OLIF technique establishes exposure with a probe and dilator which substantially limits direct visualization of the surgical field and may endanger neural and vascular structures which traverse the OLIF corridor. In some cases of severe degeneration with osteophytes, it is difficult to localize the disc space without direct visualization. The modified OLIF approach described here allows for direct microscopic visualization during dissection and exposure. Nerves, vessels, and other critical intra-abdominal structures can be easily identified and protected under direct microscopic visualization. In addition, any bleeding sources can be clearly identified stopped under direct microscopic visualization, resulting in a safer procedure.

Molinares et al. dichotomized cases into those with an Oblique Corridor (OC) (vessels and psoas were not in direct contact) and those without (11). Ng et al. classified OC into 4 grades according to the length of OC, where grade 0 was considered as surgical contraindication for OLIF because there was no space between the blood vessel and the psoas major muscle (22). The OC in cases of Grade 1 or 2 are generally not enough for spinal exposure. The establishment of an approach corridor may decrease in the right lateral decubitus position due to the relaxed psoas muscle (9, 10, 13). Although not an absolute contraindication to the T-OLIF approach, narrow OCs may require increased operative dissection to mobilize the psoas complex in order to widen the OC and allow for the surgery to be executed (22). Generally, cases with OC 0 are a contraindication for the T-OLIF approach.

Even in cases in which there is a wide enough OC on imaging, it may not be easy to expose and establish a corridor; sometimes, a very ventrally deviated psoas may preclude safe access to the lumbar spine (21). Previous studies have focused on a particular psoas variant known as the tear-drop psoas or “Mickey Mouse ears” psoas. This psoas variant is thought to preclude the lateral approach because of the ventral deviation of the lumbar plexus (22). In these cases, the OC approach will entail circumventing this bulky psoas, and this may be difficult. Moreover, the tear-drop psoas is also associated with a lateral and posterior deviation of the iliac vessels, further narrowing the OC length and increasing the risk of complications during OLIF (21). In such anatomic variants, the lumbar plexus may be over stretched or compressed against the transverse processes in order to obtain access, resulting postoperative motor or sensory deficits and hip flexion weakness. To decrease the rate of this complication, the retraction of psoas should be limited. We split the anterior portion of psoas belly along the psoas major muscle fibers in order to gain access to the corridor. With this selective splitting of the psoas, we decrease the amount of psoas muscle to be retracted, minimizing lumbar plexus stretching and compression. Unlike the traditional transpsoas lateral lumbar interbody fusion accessing the lumbar spine from a true lateral position, our modified AP-OLIF approach accesses the anterolateral aspect of the lumbar spine (Figure 6). The splitting of the psoas occurs only at the anterior portion of psoas belly instead of performing significant retraction of the psoas (Figure 6). The splitting point of the psoas is anterior to the lumbar plexus. Thus, our modified approach is within the safe working zone as defined by previously published anatomic studies (25, 26). Even if there is variability in the lumbar plexus anatomy, the plexus usually can be identified and protected under direct microscopic visualization. Electrophysiologic neuromonitoring is not mandatory.

This study has a few limitations. Firstly, this was a retrospective study and although we tried to avoid selection bias, a prospective study is preferably needed, so we hope to conduct a prospective randomized control trial to conduct a more comprehensive evaluation. Second, L2–L5 lumbar degenerative diseases were included in the study and were not the same segment; the different segments may affect the parameters, complications, and outcome analysis and evaluation.

## Conclusion

For patients with grade 1 or 2 OC or with modified Moro’s psoas classification of I and AI–AIV, the modified AP-OLIF allows for easier and safer to the lumbar spine from L1 to L5. The modified approach blends the advantages of both the true lateral transpsoas approach and the oblique approach to allow for an easier exposure that also protects the lumbar plexus and critical structures.

## Data Availability

The original contributions presented in the study are included in the article/Supplementary Material, further inquiries can be directed to the corresponding author.
